# Supplementary material for: Nucleation of Biomolecular Condensates from Finite-Sized Simulations
Source: J Phys Chem Lett. 2023 Feb 9;14(7):1748–55. doi: 10.1021/acs.jpclett.2c03512 (PMC9940850; doi:10.1021/acs.jpclett.2c03512)
Supplement: Supplementary file 2 — jz2c03512_si_002.pdf [file jz2c03512_si_002.pdf]

Dear Prof. Mennucci,

Thank you for handling our paper, in our revision we have incorporated the editorial comments and addressed the points raised by the reviewer. A detailed point-by-point response follows, where the comments are reported in black and the authors' response is reported in blue. Excerpts from the reviewed text are reported in *blue, italics*.

Editorial Changes:

1. Abstract: Shorten the abstract to 150 words or fewer.

The abstract has been reduced to < 150 words. The new abstract is:

"The nucleation of protein condensates is a concentration-driven process of assembly. When modelled in the canonical ensemble, condensation is affected by finite-size effects. Here, we present a general and efficient route to obtain ensemble properties of protein condensates in the macroscopic limit from finite-sized nucleation simulations. The approach is based on a theoretical description of droplet nucleation in the canonical ensemble and enables estimating thermodynamic and kinetic parameters, such as the macroscopic equilibrium density of the dilute protein phase, the condensates surface tension and nucleation free energy barriers. We apply the method to coarse-grained simulations of NDDX4 and FUS-LC, two phase-separating peptides with different physicochemical characteristics. Our results show that NDDX4 condensate droplets, characterised by lower surface tension, higher solubility, and faster monomer exchange dynamics than FUS-LC, form with negligible nucleation barriers. In contrast, FUS-LC condensates form via an activated process over a wide range of concentrations."

2. TOC Graphic: Please resize the TOC graphic per journal guidelines (2 in x 2 in) and move to the correct position (on the same page as the abstract).

The TOC has been resized and moved to the same page as the abstract.

3. Supporting Information: Please remove your Supporting Information from the manuscript file and upload it as a "Supporting Information For Publication" file.

The SI has been uploaded as a separate file.

4. Supporting Information: Please add full header at top of page of the Supporting Information file, which includes: Title (in title case), Full Author List, and Author affiliations (exactly as they appear in the manuscript).

The Supporting information have been reformatted as requested.

5. References: In both the main file and the supporting information, fix the style of all references to use JPCL formatting (check all references carefully). ***JPC Letters reference formatting requires that journal references should contain: () around numbers, author names, article title (titles entirely in title case or entirely in lower case), abbreviated journal title (italicized), year (bolded), volume (italicized), and pages (first-last). Book references should contain author names, book title (in the same pattern), publisher, city, and year. Websites must include date of access.

Journal names have been abbreviated and the bibliography style changed to match the ACS template.

Referee 1.

The manuscript by Lunna, Paloni et al. describes a new approach to predict thermodynamic and kinetic properties of biomolecular condensates from finite-size molecular dynamics (MD) simulations. Building on the previously proposed Modified Liquid Droplet model, the authors use a residue-level model to show how to consistently estimate macroscopic quantities, such as saturation concentration and surface tension, from simulations of droplets of less than a hundred protein chains. Moreover, the new method described in the manuscript enables to access quantitative information on nucleation kinetics using standard MD simulations in the canonical ensemble.

This is a major methodological advancement as the quantification of nucleation free energy barriers and critical nucleus sizes typically requires computationally expensive enhanced sampling methods. The approach demonstrated in this work should be widely useful for studying the phase separation of biomolecular systems since both thermodynamics and kinetics analyses are crucial for a complete understanding of the process. Whereas the text is overall clear and concise, additions and clarifications would be helpful to further guide the reader.

We thank the reviewer for their positive response to our contribution, and their recommendation to accept the paper with minor revisions.
In the following, we have answered all technical points raised and edited our manuscript accordingly.

1. It would be useful if the comparison between slab and nucleation results also included the surface tension. The estimate from direct-coexistence simulations could be obtained using the pressure tensor, as for example in Tejedor et al. 2021, DOI: 10.1016/j.bpj.2021.11.003. I believe this would further strengthen the validation of the proposed method.

We have extended the slab simulations and applied the methods described in the paper of Tejedor et al. and highlighted by the reviewer to estimate the surface tension from the pressure tensor. The estimates obtained are in excellent agreement with those obtained from the fitting of the modified liquid droplet free energy model. We obtain for NDDX4, 0.125 ± 0.099 [mN/m] (compared to a surface tension of 0.101 ± 0.06 mN/m from nucleation simulations) and for FUS-LC, 0.291 ± 0.026 [mN/m] (compared to a surface tension of 0.37 ± 0.11 mN/m from nucleation simulations).
In both cases the estimates obtained with the two methods agree within statistical uncertainties. The uncertainties for the surface tensions evaluated from slab simulations was obtained by dividing the trajectories into two blocks. The following sentence has been added to the text to describe the comparison,

The surface tension σ is estimated to be 0.37 $\pm$ 0.11 mN/m, for FUS-LC and 0.101 $\pm$ 0.06 mN/m for NDDX4, reflecting the higher hydrophilic character of NDDX4 compared with FUS-LC. These estimates are in excellent agreement with surface tension estimates computed from slab simulations\cite{tejedor2021biophysj}, yielding 0.125 ± 0.099 and 0.291 ± 0.026 mN/m for NDDX4 and FUS-LC, respectively.

2/3. On page 2, left column, several parameters are introduced as molar densities but they are expressed in both nm^{-3} and mg/ml later in the text. It would be helpful if the same units were used throughout, preferably molar concentrations or mg/ml to facilitate the comparison with reference literature values for NDDX4 and FUS LC.
In the legends of Fig 1 F and G, Fig 2 C, and in several SI figures (legends and figure titles), the units of the densities should be specified.

All of the densities/concentrations in the main paper and SI figures are now expressed in the unit of mg/ml for better comparison with experiments. Similarly, the main text and supporting material are modified so that the unit of mg/ml is now used consistently during

any discussion of densities/concentrations. In Table I, II and S1, densities/concentrations are expressed in both units of mg/ml and nm^{-3}.

4. The nomenclature used for the densities would be clearer if explained in reference to Eq 2, that is rho_d^o= rho_d^{n=0} and rho_d^*= rho_d^{n=n*}. On page 2, line 57, left column, it would be helpful to clarify that rho_d^o is the total protein density, N/V, since the definition as “the density of the dilute phase at n=0” might be unambiguous only in relation to Eq 2, which is introduced later in the text.

The quantity rho_d^o has been redefined as suggested by the reviewer.

5. It would be useful to specify, in the caption or in the main text, the parameters used to calculate the free energy profiles shown in Fig 1 E.

The caption of Figure 1E, has now been revised as follows:

“Coarse-grained modelling of the nucleation of bio-molecular condensates in the NVT ensemble. A-B) Coarse-grained model of the NDDX4 and FUS-LC chains. C) Example of supersaturated homogeneous dilute phase configuration. D) Example of a steady-state configuration containing a stable, condensed phase droplet. E) Example free energy profiles associated with the nucleation of condensed phase droplets, obtained by keeping the total number of peptide chains constant while increasing the total volume. The variable n represents the number of chains in the dense phase; the origin corresponds to a homogeneous dilute phase; the local maximum at small n corresponds to the critical nucleus size; and the local minimum at large n corresponds to the steady-state droplet size. The free energy profiles are obtained using Eq. 1 with σ=0.182 mN/m, ρ_d^*=1 mg/ml, a total number of 187 chains, and a box-length ranging from 105 to 68 nm. F, G) Steady-state droplet radial density profiles obtained from simulations performed in different box sizes for NDDX4 (F) and FUS-LC (G).”

6. On page 4, line 34, left column, “we performed for 1 μs” could be replaced with “we performed simulations for 1 μs”.

The sentence has been corrected as suggested.

7. On page 4, line 3, right column, it would be helpful to cite, right after “experimental results”, the papers reporting the dense-phase protein concentrations of NDDX4 and FUS LC.

References to the experimental work are now included as the reviewer suggested.

8. On page 5, line 58, left column, it is not completely clear what the word “these” refers to. Please consider moving the sentence on page 5, line 31, right column, to the beginning of the paragraph about Fig 2 to state what Fig 2 demonstrates before delving into its description.

Both sentences have been revised as suggested, to improve clarity:

Fig. 2 demonstrates how the proposed methodology provides a direct route to extract the macroscopic thermodynamic parameters that enable such characterisation from simple finite-sized simulations. Examples of finite-size free energy profiles, obtained for NDDX4 and FUS-LC, are provided in Fig. 2A at one of the total densities simulated. Nucleation free energies in the macroscopic limit are instead reported for all densities investigated in Fig. 2B.

9. It would be useful to explicitly mention and comment on Fig 2 A in the text.

As detailed in the previous answer, a reference to Fig. 2A has been added.

10. It would add to the clarity of Fig 2 A to use different line styles for the different system sizes and to report the box side lengths, L, in the legend, as in Fig 1 F and G.

Figure 2A is modified to include different line styles for different bulk density/box size combinations to better identify different simulation set-ups.

11. In the caption of Fig 2 B, "for the same peptide" could be replaced with "for each protein".

The sentence has been amended as indicated.

12. In Fig 2 B, the readability of the figure would benefit from reporting in the legend all the total protein densities (or preferably protein concentrations) corresponding to the different curves (solid, dashed, and dot-dashed).

Figure 2B is now modified to include all of the protein concentrations in units of mg/ml.

13. On page 6, left column, it is unclear how the dynamics of the exchange of chains between the coexisting phases, which is obtained using Markov state models, contributes to explain the different phase behaviour of NDDX4 and FUS LC. The authors could make the relationship with the nucleation results more evident in the text.

This aspect has been clarified in the following sentence:

At steady state, nucleation simulations provide an extensive sampling of the dynamic exchange of monomers between the condensed and dilute phases. We can exploit this to obtain a quantitative description of the single chain exchange dynamics, with the aim of complementing the collective information captured by nucleation free energy profiles. To this aim, we analyse the dynamics of single-chain exchange between the condensed and dilute phases using Markov state models (MSMs). The details of the MSM construction and analysis are reported in Supporting Information…

14. In the caption of Fig 3, please clarify what solid and dashed lines show in the insets.

The explanation of the solid and dashed lines is now added to the caption.

The white region represents a \emph{nominally supersaturated} portion of parameter space; here finite-size effects mean that, $F(n)$ (represented as a solid line in the insets) is a monotonically increasing function of n, even if the corresponding free energy in the macroscopic limit (represented as a dashed line) permits a critical nucleus. In these conditions condensation is inhibited by the confinement (see inset III).

15. In Fig S5, please specify the units in the axes' labels of the inset.

The unit for the radii (nm) in the upper inset of Fig. S5 is now added.

16. Please specify in the SI the cutoff at which Lennard-Jones and Debye-Hückel interactions were truncated and whether nonbonded interactions were modified by a shift function.

Interactions were truncated, not shifted with a cutoff of 4 nm. This information has been added to the SI in the section dedicated to the description of the coarse grained forcefield as follows:

"All non-bonded interactions were truncated with a cut-off of 4 nm."

17. In Fig S11, please clarify in the caption which values are indicated by the dashed horizontal lines. I would also suggest using a log-scale for the y-axis to facilitate the comparison between the dilute-phase concentrations.

We prefer to maintain the y axis on a linear scale in order to avoid misrepresenting the agreement between the density of the condensate obtained from the slab method and experiments. We have, however, complemented the figure by reporting the experimental and simulation estimates to enable a better assessment of the agreement of the dilute phase equilibrium densities.

18. I would like to encourage the authors to make openly available the in-house analysis packages and scripts used for the analyses described in the paper.

The code used to analyse the trajectories is available on github at https://github.com/mme-ucl/confined_LLPS. This information has been added to the paper, in the Supplementary Information section.

Jupyter notebooks used in the data analyses are available for download at https://github.com/mme-ucl/confined_LLPS.
